# Deciphering the etiology of undiagnosed ocular anomalies along with systemic alterations in pediatric patients through whole exome sequencing

**DOI:** 10.1038/s41598-024-65227-6

**Published:** 2024-06-22

**Authors:** Miriam E. Reyna-Fabián, Liliana Fernández-Hernández, Sergio Enríquez-Flores, David Apam-Garduño, Carolina Prado-Larrea, Go Hun Seo, Rin Khang, Vianney Cortés-González

**Affiliations:** 1 Laboratorio de Biología Molecular, Subdirección de Investigación Médica, Instituto Nacional de Pediatría, Mexico City, Mexico; 2 Laboratorio de Biomoléculas y Salud Infantil, Instituto Nacional de Pediatría, Mexico City, México; 3 Escuela Superior de Medicina, Instituto Politécnico Nacional, Mexico City, México; 4 Departamento de Glaucoma, Asociación Para Evitar la Ceguera en México, Mexico City, México; 5 Medical Genetics Division, 3Billion, Inc., Seoul, South Korea; 6 Departamento de Genética, Asociación Para Evitar la Ceguera en México, Vicente García Torres No. 46 Barrio San Lucas, Coyoacán, C.P. 04030 Mexico City, México; 7 Facultad de Medicina, Universidad Nacional Autónoma de México, Mexico City, Mexico

**Keywords:** Genetics, Molecular biology

## Abstract

Inherited and developmental eye diseases are quite diverse and numerous, and determining their genetic cause is challenging due to their high allelic and locus heterogeneity. New molecular approaches, such as whole exome sequencing (WES), have proven to be powerful molecular tools for addressing these cases. The present study used WES to identify the genetic etiology in ten unrelated Mexican pediatric patients with complex ocular anomalies and other systemic alterations of unknown etiology. The WES approach allowed us to identify five clinically relevant variants in the *GZF1*, *NFIX*, *TRRAP*, *FGFR2* and *PAX2* genes associated with Larsen, Malan, developmental delay with or without dysmorphic facies and autism, LADD1 and papillorenal syndromes. Mutations located in *GZF1* and *NFIX* were classified as pathogenic, those in *TRRAP* and *FGFR2* were classified as likely pathogenic variants, and those in *PAX2* were classified as variants of unknown significance. Protein modeling of the two missense *FGFR2* p.(Arg210Gln) and *PAX2* p.(Met3Thr) variants showed that these changes could induce potential structural alterations in important functional regions of the proteins. Notably, four out of the five variants were not previously reported, except for the *TRRAP* gene. Consequently, WES enabled the identification of the genetic cause in 40% of the cases reported. All the syndromes reported herein are very rare, with phenotypes that may overlap with other genetic entities.

## Introduction

Inherited and developmental eye diseases are quite diverse and numerous, including but not limited to microphthalmia, anophthalmia and coloboma spectrum (commonly referred to as MAC), congenital cataracts, aniridia, anterior segment dysgenesis anomalies, primary congenital glaucoma, optic nerve abnormalities, and degenerative entities such as corneal dystrophies, vitreoretinopathy, and retinal dystrophies, among others. Although all of these diseases are rare, they are important causes of blindness and visual impairment in childhood, having a significant emotional, social and economic impacts on individuals, their families, and society. Among the estimated 1.4 million children worldwide who are visually impaired^1^, a substantial number are predisposed to comorbidities arising from systemic complications associated with their primary ocular condition. Inherited retinal dystrophies represent a significant etiology of progressive vision loss in the pediatric population, affecting approximately 1 in 3000 patients^2^. Cataracts constitute a predominant cause of blindness, contributing to half of all cases and one-third of global visual impairment cases. The incidence of congenital cataracts, a leading cause of treatable blindness worldwide, varies between 12 and 136 per 100,000 births, with hereditary factors implicated in approximately 8.3 and 25% of congenital or infantile cataract cases^3,4^. Additionally, anophthalmia and microphthalmia are the most severe developmental eye disorders and are frequently responsible for severe visual impairment in children, accounting for approximately 3–12% of visual impairment in children^5^. All these individuals face a higher mortality rate during childhood than do their healthy counterparts^2^.

The eye represents a distinct target organ for gene therapy, encompassing both the anterior and posterior segments, specifically the cornea and retina, respectively. This uniqueness stems from the immunoprivileged status conferred by the blood-ocular barrier, enabling direct visualization, access, and localized treatment of target tissues. Additionally, the inherent advantage of simultaneous control provided by the contralateral eye enhances the precision and efficacy of therapeutic interventions^6,7^. Luxturna (voretigene neparvovec-rzyl) represents a major milestone in ocular gene therapy advancement for patients with biallelic variants in the *RPE65* gene^8^. Hence, the elucidation of gene variants linked to ophthalmologic disorders is imperative for understanding the underlying pathophysiology. Such insights serve as pivotal groundwork for the formulation of novel therapeutic modalities or for the repurposing of existing drugs.

The implementation of massive parallel sequencing is currently a widely used strategy for identifying responsible genotypes in monogenic entities with allelic and locus heterogeneity. In particular, gene‒target panels designed for nonsyndromic and syndromic variants have been the key for the diagnosis of inherited eye disorders due to their high throughput and low cost. In this sense, Patel et al. published the results of The Oculome Panel test, which revealed 429 genes associated with ocular disease. The test was conducted on a cohort of 224 patients who presented with various eye disorders^9^. The molecular detection rate was 24.5% (68 of 277 samples), with variability depending on the clinical condition; this rate was higher for retinal dystrophies and congenital cataracts (42.8–88.9%) and lower for MAC and anterior segment dysgenesis, including glaucoma (8.2–24.8%). In addition, research by Haer-Wigman et al. also showed the usefulness of combining gene panel and WES strategies in the ophthalmic area when studying 266 Dutch patients with different inherited eye disorders^10^. First, they performed a vision gene panel analysis with a diagnostic yield of 49%; subsequently, they conducted a whole exome sequencing (WES) study in those cases in which the customized gene panel was negative; this latter strategy added 2% to the diagnosis detection rate.

Currently, there are several WES studies addressing rare diseases, but little has been documented on its use in patients with complex ocular anomalies associated with other systemic manifestations. Deciphering the etiology in such patients may be difficult due to variable expressivity and/or genetic heterogeneity or non-Mendelian origin (e.g., teratogens or complex inherited diseases). Therefore, the present study harnesses the power of WES as an invaluable molecular tool to identify the genetic etiology in ten pediatric patients with complex ocular anomalies and other systemic alterations. To the best of our knowledge, this study is one of the rare few to conduct a meticulous molecular investigation into the co-occurrence of these manifestations, an exceptional contribution to the scientific landscape, particularly within the Latin American region.

## Results

### Patients

**Figure 1:**
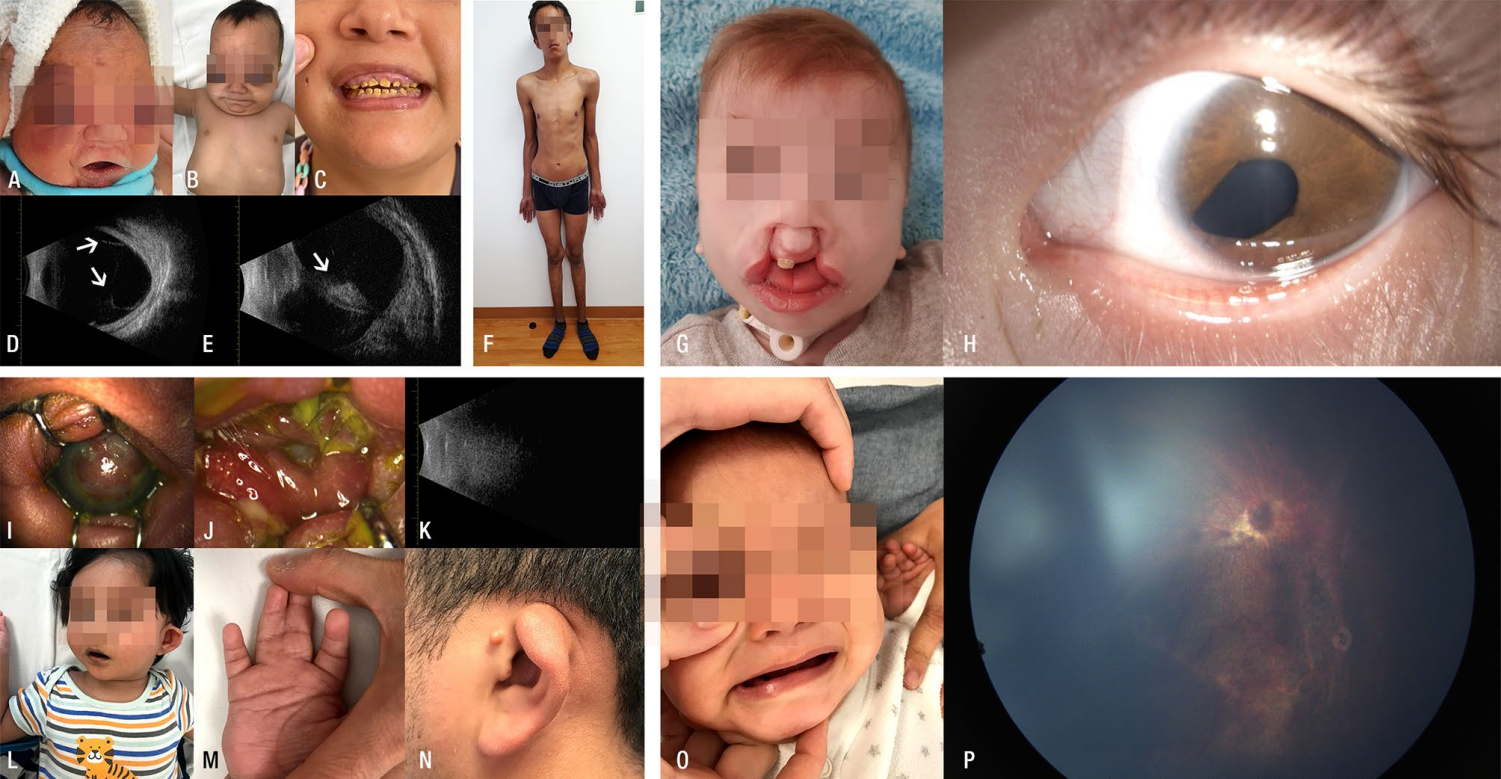
Clinical features of the five patients with clinically relevant variants identified by WES. (**A**–**E**) Patient with JLSM syndrome exhibits bilateral buphthalmos, palpebral edema and ecchymosis (A, B); amelogenesis in the proband´s sister (C); b-mode ocular ultrasound with axial length of 24.17 mm and anterior posterior vitreous detachment (D) followed by retinal detachment in the OD (**E**). (**F**) Patient with Malan syndrome who presented dolichostenomelia, pectus excavatum, and a prominent forehead. (**G**,**H**) Patient with DEDDFA displays features of medial cleft lip and palate and iris coloboma. (**I**–**N**) Patient with LADD1 syndrome with corneal opacity in the OD (I) and clinical anophthalmos in the OS (**J**). B-mode ocular ultrasound revealed no ocular structures (**K**), facial dysmorphism (**L**), syndactyly (**M**), and abnormal pinna morphology in the affected father (**N**). (**O**,**P**) Fundoscopy revealed optic nerve aplasia in OS in a patient with PAPRS.

We included ten unrelated Mexican pediatric patients who had ocular anomalies and several systemic manifestations of unknown etiology (Fig. 1, Table 1). All patients were evaluated by an ophthalmologist and medical geneticist at the Asociación para Evitar la Ceguera en México, Mexico City. All patients and their families received genetic counseling, and parents or legal guardians signed an informed consent form before carrying out the molecular study, as well as an iconographic informed consent form for the collection and use of clinical photographs.

**Table 1:** Ocular and systemic alterations observed in the ten studied patients.

Patient	Age	Sex	Ophthalmological findings	Systemic manifestations	Additional studies
1	3d	M	OU: palpebral edema (HP:0100540), buphthalmos (HP:0000557), ecchymosis (HP:0031364), corneal opacity (HP:0007957), congenital glaucoma (HP:0008007), abnormality iris morphology (HP:0000525), retinal detachment (HP:0000541)	Disproportionate short stature (HP:0003498), hypotonia (HP:0001252), patent foramen ovale (HP:0001655)	Conventional karyotype: normal Brain MRI: normal
2	13 y	M	OU: Deeply set eyes (HP:0000490), exotropia (HP:0000577)	Severe intellectual disability (HP:0010864), dolichocephaly (HP:0000268), triangular face (HP:0000325), prominent forehead (HP:0011220), tooth malposition (HP:0000692), pectus excavatum (HP:0000767), dolichostenomelia (HP:0001519), arachnodactyly (HP:0001166)	Conventional karyotype: 46,XY Brain computed axial tomography: normal
3	2.5 y	M	OS: iris coloboma (HP:0000612)	Hypotonia, neurodevelopmental delay (HP:0012758), seizures (HP:0001250), microcephaly (HP:0000252), highly arched eyebrows (HP:0002553), medial cleft lip and palate (HP:0008501), bilateral cryptorchidism (HP:0008689)	Brain MRI: Dandy Walker disease, thinning of the corpus callosum Conventional karyotype and aCGH tests: normal
4	2.1 y	M	OD: corneal opacity (HP:0007957), microphthalmia (HP:0000568) OS: anophthalmia (HP:0000528)	Hypotonia, neurodevelopmental delay (HP:0012758), cupped ear (HP:0000378), low-set ears (HP:0000369), severe hearing impairment (HP:0012714), atresia of the external auditory canal (HP:0000413), conical tooth (HP:0000698), syndactyly (HP:0001159), small nail (HP:0001792)	Kidney USG: normal Metabolic neonatal screening: normal
5	4m	F	OD: microphthalmia OS: microcornea, iris coloboma, cataract (HP:0000518) OU: optic nerve aplasia (HP:0008058), retinal detachment (HP:0000541)	Hypotonia, neurodevelopmental delay (HP:0012758)	Metabolic neonatal screening, kidney USG, and aCGH tests: normal. Brain MRI: abnormal
6	11y	F	Polymorphous posterior corneal dystrophy (HP:0007915)	No extraocular findings	AD (familial case with 1st and 2nd relative’s degree affected)
7	3y	M	Apparently congenital stromal corneal dystrophy	No extraocular findings	Sporadic
8	3y	M	OD: Maculopathy (HP:0011504)	Global developmental delay (HP:0001263), microcephaly (HP:0000252), seizures (HP:0001250), polysplenia (HP:0001748), cryptorchidism (HP:0000028)	Consanguinity TORCH screen, conventional karyotype and aCGH tests: normal. Brain MRI: cebocephaly, absence of lateral ventricle floor, dilatation of cerebral ventricles, hypoplasia of the corpus callosum, encephalomalacia, calcifications of frontal, temporal, and occipital lobes
9	15 y	M	Congenital cataract (HP:0000519), Keratoconus (HP:0000563)	Intellectual disability (HP:0001249), pain insensitivity (HP:0007021)	Sibling with hypoplasia of the optic nerve The patient´s mother had gestational diabetes
10	10y	M	OS: Anophthalmia (HP:0000528)	Single central incisor (HP:0006315), scoliosis (HP:0002650)	Spinal X-ray: vertebral malformations at T5 and T6 level

aCGH: Microarray-based Comparative Genomic Hybridization; AD: autosomal dominant; d: days; F: female; M: male; m: months; MRI: magnetic resonance imaging; OD: right eye; OS: left eye; OU: both eyes; TORCH: toxoplasmosis, others (syphilis, hepatitis B), rubella, cytomegalovirus, herpes simplex; USG: ultrasonography; y: years.

Of the 10 patients included in this study, 8 were males and 2 were females, and the mean age was 6.3 years (range: 3 days -15 years). According to clinical data, two patients had an affected first-degree relative with an autosomal dominant inheritance pattern; thus, they were considered familial cases (Patients 4 and 6). In addition, two patients had a history of inbreeding or consanguinity (Patients 1 and 8), which suggested an autosomal recessive pattern (Table 1).

### Molecular analyses

**Figure 2:**
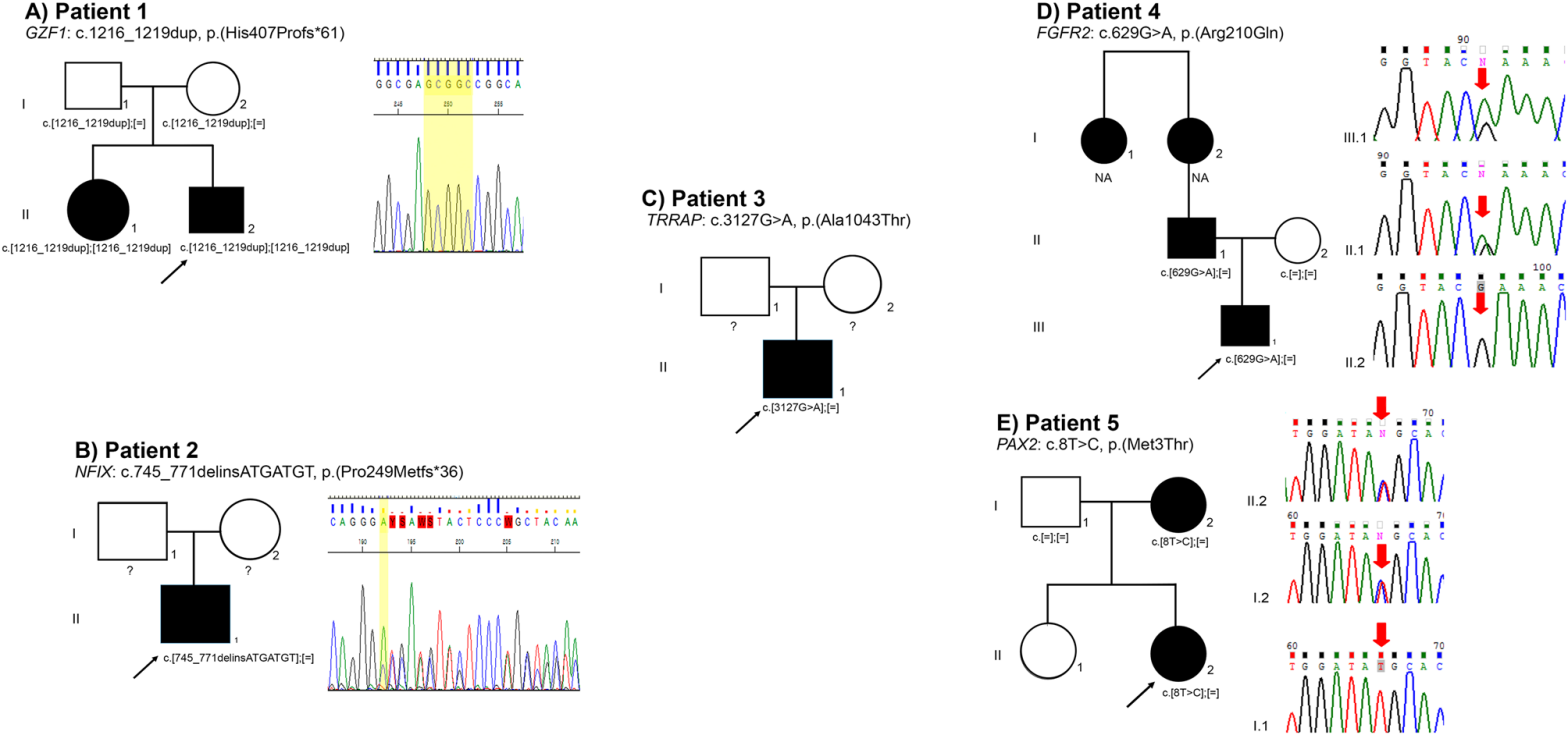
Pedigrees and electropherograms of the five patients with clinically relevant variants identified by WES. (**A**) *GZF1* partial sequence showing the c.1216_1219dup, p.(His407Profs*61) variant (shaded in yellow) from Patient 1. The pedigree shows both parents with a heterozygous mutation, and the proband's sister is affected by a homozygous mutation. (**B**) The *NFIX* partial sequence shows the c.745_771delinsATGATGT, p.(Pro249Metfs*36) variant in the heterozygous state from Patient 2. (**C**) *TRRAP* pedigree showing affected Patient 3 with a heterozygous variant. (**D**) The *FGFR2* partial sequence shows the c.629G>A, p.(Arg210Gln) variant from Patient 4 (III.1) and her father (II.1). The mother of Patient 4 has the wild-type allele (II.2). The pedigree additionally indicated that the paternal grandmother and aunt were affected. (**E**) *PAX2* partial sequence showing the c.8T>C, p.(Met3Thr) variant from Patient 5 and her mother. NA: not available for the molecular study);?: the parents did not consent to the molecular test.

The median read depth for the WES study was 255X (range 185X–413X), with 98.9% coverage. This molecular approach allowed us to identify clinically relevant variants in the *GZF1* (MIM*613842), *NFIX* (MIM*164005), *TRRAP* (MIM*603015), *FGFR2* (MIM*176943) and *PAX2* (MIM*167409) genes in five patients (Patients 1–5; Figs. 1 and 2, Table 2). According to the ACMG/AMP variant classification, mutations located in the *GZF1* (c.1216_1219dup) and *NFIX* (c.745_771delinsATGATGT) genes were classified as pathogenic variants (PV), while mutations in *FGFR2* (c.629G>A) and *TRRAP* (c.3127G>A) were classified as likely pathogenic variants (LPV) and mutations in *PAX2* (c.8T>C) were classified as variants of unknown significance (VUS). Table 2. Neither PV located in *GZF1* nor *NFIX* has been reported in healthy or patient public databases (last accessed March 22th, 2024), such as the Genome Aggregation Database v4.0 (https://gnomad.broadinstitute.org/), the Human Gene Mutation Database v.2022.2 (http://www.hgmd.cf.ac.uk/ac/index.php), and the Leiden Open Variation Database v.3.0. (LOVD, www.lovd.nl/), dbSNP Build152 (https://www.ncbi.nlm.nih.gov/projects/SNP), and ClinVar v1.0 (https://www.ncbi.nlm.nih.gov/clinvar/), and they were also absent in the literature. The five genetic variants identified herein were submitted to the LOVD Database v.3.0 (*GZF1*: https://databases.lovd.nl/shared/individuals/00402795; NFIX: https://databases.lovd.nl/shared/individuals/00402809; *TRRAP*: https://databases.lovd.nl/shared/variants/0000839638#00025389; *FGFR2*: https://databases.lovd.nl/shared/individuals/00402813; and *PAX2*: https://databases.lovd.nl/shared/individuals/00402842). The syndromes associated with these genotypes are joint laxity, short stature, and myopia (JLSM [MIM: 617662], Fig. 1A–E), Malan syndrome (also known as SOTOS2 syndrome [MIM: 614753], Fig. 1F), developmental delay with or without dysmorphic facies and autism (DEDDFA [MIM: 618454], Fig. 1G,H), lacrimoauriculodentodigital syndrome 1 (LADD1 [MIM: 149730], Fig. 1I–N), and papillorenal syndrome (PAPRS [MIM: 120330], Fig. 1O,P).

**Table 2:** Genetic information of the five clinically relevant variants identified by WES.

Patient	Gene	Variant	Genotype	Mean depth	ACMG/AMP classification	Reports in literature/reports in public databases	Phenotype	Inheritance
1	*GZF1*	NM_001317012.1:c.1216_1219dup p.(His407Profs*61)	Homo	265X	Pathogenic [PVS1, PP5, PM2]	NPR/LOVD v.3.0: GZF1_00402795^a^	Joint laxity, short stature and myopia (JLSM)	AR (inbreeding) sister homozygous
2	*NFIX*	NM_001271043.2:c.745_771delinsATGATGT p.(Pro249Metfs*36)	Hetero	208X	Pathogenic [PVS1, PM2, PP3]	NPR/LOVD v.3.0: NFIX_00402809^a^	Malan syndrome (known as SOTOS2)	Assumed de novo
3	*TRRAP*	NM_001375524.1:c.3127G>A p.(Ala1043Thr)	Hetero	203X	Likely pathogenic [PM1, PM2, PM5, PP3, PP5]	Cogné et al.^17^/dbSNP: rs1562945106, ClinVar v1.0: RCV000785654.1, LOVD v.3.0: TRRAP_00402812^a^	Developmental delay with or without dysmorphic facies and autism (DEDDFA)	Assumed de novo
4	*FGFR2*	NM_022970.3:c.629G>A p.(Arg210Gln)	Hetero	185X	Likely pathogenic [PP1, PM1, PM2, PP3, PP5]	NPR/dbSNP: rs2134609287, gnomAD v.4.0: 10-121538711 (AF:6.195e−7), LOVD v.3.0: FGFR2_00402813^a^	Lacrimoauriculodentodigital Syndrome 1 (LADD1)	AD (paternal inheritance)
5	*PAX2*	NM_003990.4:c.8T>C p.(Met3Thr)	Hetero	413X	VUS [PM2, PP2]	NPR/dbSNP: rs754968736, gnomAD v4.0: 10-100746268 (AF:0.000002737), LOVD v.3.0: PAX2_00402842^a^	Papillorenal syndrome (PAPRS)	AD (maternal inheritance)

AD: autosomal dominant; AR: autosomal recessive; Homo: homozygous; Hetero: heterozygous; NPR: not previously reported; VUS: variant of uncertain significance.

*Target covered > 10X, apparently de novo was mentioned when molecular study in parents was not performed.

^a^Individual identification as referenced in the LOVD database v3.0.

### Protein modeling of the two missense FGFR2 and PAX2 variants

**Figure 3:**
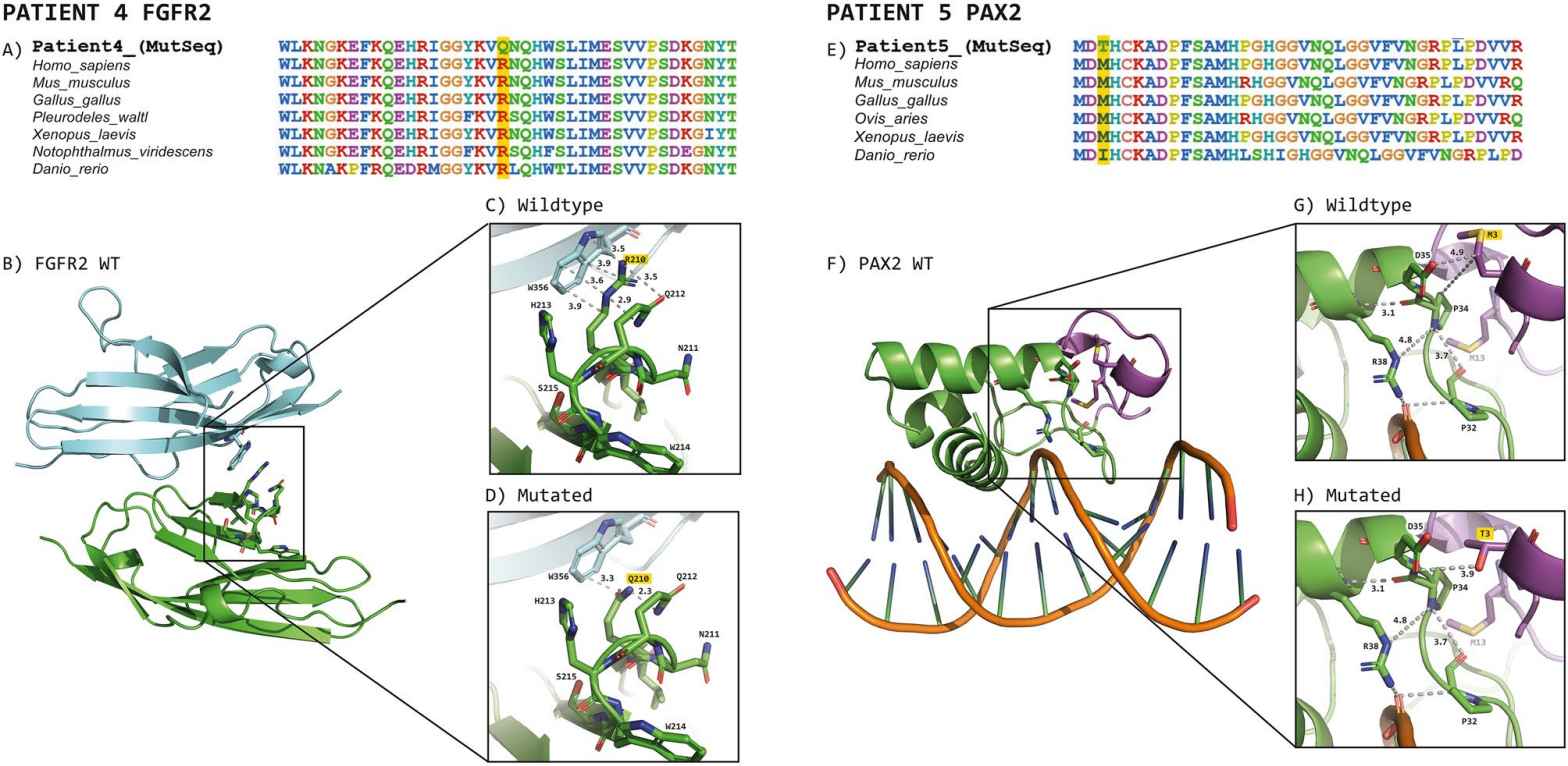
Sequence alignment and modeling of FGFR2 and PAX2 structures in loop and ribbon formats. (**A**,**E**) FGFR2 and PAX2 amino acid alignment of vertebrate organisms; the mutated amino acid position is highlighted in yellow. (**B**) 3D structures of FGFR2 (green) in complex with FGF1. Notably, the interaction between the two subunits is mediated in part by the aminoacyl residue Arg210. (**C**) Which corresponds to the increase in Arg210 and adjacent amino acids, is the main interaction distance (in Å) of this zone. (**D**) represent the same zone but with the substitution of Asn (modeled in silico), it is evident that the mutation caused the loss of mediated interactions in both FGFR2 and FGF1. (**F**) shows the 3D model of PAX2 (green), which was obtained from the crystallographic structure of PAX5. The region in purple corresponds to the first 18 aminoacyl residues not contained in the template structure. In general, the protein extensively interacts with some regions of the DNA. (**G**,**H**) Zoomed view of the N-terminus of PAX2. (**G**) The interaction distances between Met3 and other aminoacyl residues can be observed, while H) The substitution of Met3 with Thr (Thr3) is shown (modeled in silico). Figures were modeled with PyMOL (The PyMOL Molecular Graphics System, Version 2.5.3, Schrödinger, LLC).

To examine the probable structural consequences of the two missense variants that were classified as LPV [*FGFR2*: c.629G>A or p.(Arg210Gln)] and VUS [*PAX2*: c.8C>T or p.(Met3Thr)], in silico molecular modeling of the FGFR2 and PAX2 proteins was carried out (Fig. 3).

FGFR2 serves as a pivotal cell-surface receptor that orchestrates critical functions, including the regulation of cell proliferation, migration, differentiation, angiogenesis, and tissue repair^11^. Recognizing its vital roles, we embarked on modeling the 3D structure with the corresponding mutation. According to the 3D FGFR2 model (Fig. 3B), the amino acid residue Arg210 is located in the zone of interaction (interface) between the FGFR2 and FGF1 subunits (Fig. 3C). At this interface, crystallographic contacts were identified between this aminoacyl residue and the adjacent subunit, which specifically interact with Trp356 of the FGF1 subunit via cation-π interactions. They are strong interactions between positively charged entities and the π electron cloud of aromatic groups that contribute to protein stability within or between subunits^12^. By in silico modeling of the p.(Arg210Gln) mutation, we detected that the Arg-by-Gln substitution caused a significant loss of important contacts between both subunits, which was originally mediated by Arg210 (Fig. 3D).

On the other hand, it has been reported that PAX2 is a transcription factor related to renal cell differentiation^13^, as well as to the development of the urogenital tract^14^ and the central nervous system^15^. According to the generated PAX2 3D model (Fig. 3F), it interacts with the DNA molecule at its N-terminal end. According to this model, Met3 is located relatively close to the DNA, interacting with the amino acid residues Pro34 and Asp35 (Fig. 3G). In the 3D structure, it is predicted that the drastic change from Met to Thr affects the potential interactions in such mutation zones (Fig. 3H), which would diminish the protein affinity to DNA.

## Discussion

The WES genomic testing conducted in this cohort of ten patients with complex ocular and systemic manifestations yielded a diagnosis in 40% of the patients, which is slightly similar to the reported diagnostic yield in the literature for Mexico (47%)^16^. Among the three disease-causing genotypes identified in Patients 1–3, the LPV of *TRRAP* p.(Ala1043Thr), identified in Patient 3, is the only genotype previously reported in five individuals with syndromic intellectual disability (ID) with malformations. This missense variant, the most recurrently observed variant among individuals with neurodevelopmental disorders and a *TRRAP* mutation (5/24), was classified as likely pathogenic by Cogné et al. based on their phenotypic and molecular findings^17^. To date, the ClinVar database reports this variant with conflicting interpretations of pathogenicity (variant ID: VCV000634849.4, accessed July 22th 2023). In the present study, we support the pathogenicity of this variant based upon the following: (a) the phenotype of Patient 3, which correlates with the other five patients bearing the same p.(Ala1043Thr) *TRRAP* mutation; (b) assumed to be de novo with no family history of the disease, Fig. 2C (healthy parents did not consent to be tested via Sanger sequencing), which is consistent with other reports in which the majority of variants in this syndrome have been documented as de novo (N = 23/24), with only one reported case of germline mosaicism (N = 1/24)^17^; (c) the amino acid evolutionary conservation of *TRRAP,* as it is among the top five genes intolerant to missense variation^18^; (d) variant deleterious prediction sustained by most in silico software tools such as CADD score^17^, Mutation Taster (disease causing, prob: 0.9999)^19^, MutPred2 (score: 0.640, probability: 0.05, *P*-value: 0.04)^20^, FATHMM-MKL (Pathogenic Moderate, Coding score:0.9944)^21^; SIFT (tolerated, score 0.27)^22^, PolyPhen-2 (benign, HumVar score:0.066 [sensitivity: 0.92; specificity: 0.65])^23^, in silico predictors, provide evidence at the supporting level or, in some cases, at the moderate level for pathogenicity classification of genetic variants. Interestingly, this residue is located in a highly mutable CpG site and in an uncharacterized TRRAP domain that clusters other pathogenic variants and may have a potentially novel specific function^17^. The clinical findings observed in our patient are consistent with the high genotype–phenotype correlation observed for this gene.

The other two frameshift *GZF1* p.[His407Profs*61] and *NFIX* p.[Pro249Metfs36] pathogenic variants causing JLSM (Patient 1) and Malan syndrome (Patient 2) are novel genotypes. The majority of patients with JLSM syndrome exhibit autosomal dominant inheritance, attributed to mutations in the *FLNB* gene; however, the *CHST3, B4GALT7* and *GZF1* genes have recently been recognized as responsible for the autosomal recessive (AR) pattern^24–26^. The *GZF1* gene contains an N-terminal BTB/POZ domain and ten tandemly repeated C2H2 zinc-finger motifs. Patient 1 harbors the *GZF1* p.[His407Profs*61] homozygous PV located at the C2H2 zinc-finger motif 4, an important region for efficient DNA binding^27^. To date, there are only two reports associating *GZF1* truncating variants with AR-JLSM syndrome in seven patients from three unrelated Saudi and Chinese families^26–28^. The ocular manifestations in those patients were severe myopia, glaucoma, retinal detachment, and iris and retinal coloboma; some of them were legally blind, and all of them showed mild articular involvement.

In the present study, Patient 1 exhibited a severe ocular phenotype, primarily characterized by palpebral edema, ecchymosis, buphthalmos, abnormalities in iris morphology, and congenital glaucoma with a detrimental progression resulting in retinal detachment after micropulse transscleral cyclophotocoagulation, followed by subsequent *phthisis bulbi* (Table 1, Fig. 1A–E); nevertheless, he did not have joint involvement. Since some traits, such as scoliosis, joint laxity and hearing loss, were reported as age-dependent manifestations in previous studies, it is feasible that Patient 1 could develop these manifestations later in his life.

The proband´s 16-year-old sister had high myopia [autorefraction in right eye (OD): − 12.00 = − 2.50 × 155°; left eye (OS): − 10.50 = − 2.75 × 25°], with an intraocular pressure (IOP) of 15 mm Hg in the OD and 16 mm Hg in the OS. The horizontal corneal diameter was 9.5 mm in the OD and 9.7 mm in the OS (corneal diameter: ± standard deviation of 11.71 ± 0.42 mm horizontally^29^), and there was corectopia in both eyes (OU). Fundoscopy revealed a choroidal fundus in the OU with an atrophic hole in the OS and a peripheral retina with microcystic degeneration. She also had short stature (Z = − 4.86), hypodontia, amelogenesis imperfecta, short neck, clubfoot, skin syndactyly between the second and third fingers, hearing loss and hip dysplasia (Fig. 1C). We further confirmed the presence of the homozygous p.[His407Profs*61] *GZF1* variant in the proband's sister, as well as its presence in both parents with a heterozygous genotype, using direct Sanger sequencing. Consequently, we categorized this case as familial, following an autosomal recessive (AR) inheritance pattern (Fig. 2A).

Moreover, it was demonstrated that the GZF1 protein is highly expressed in the adult heart, skeletal muscle, and kidney^30^, but until now, no Larsen patients have shown structural or functional anomalies in skeletal or kidney tissues. However, the proband’s sister showed a skeletal dysplasia phenotype, which could be the result of other *GZF1* pathogenic mechanisms that are still unknown. We additionally excluded any congenital heart disease through an echocardiogram study in Patient 1, consistent with Zeng et al*.* findings of one Larsen patient with Tetralogy of Fallot^28^. It is noteworthy that this represents the third report linking *GZF1* mutations to AR Larsen syndrome, with the first documented patient in the Mexican population. These findings highlight the rarity of this condition worldwide.

On the other hand, haploinsufficiency of *NFIX* caused by whole-gene deletions or heterozygous point mutations is causative of Malan syndrome^31^ (MIM#614753), which is characterized by overgrowth, dysmorphic facial features, intellectual disability and behavioral problems^32^. Recognition of these patients based on clinical manifestations could be challenging because disease severity is tremendously variable and could resemble Marfan or Marfan-like, Marshall-Smith, Weaver or Sotos syndromes. In an endeavor to delineate the Malan phenotype, Priolo et al*.* compared 80 patients and concluded that the consistent manifestations included overgrowth (height > 2 SDS in infancy and in half of affected adults), facial dysmorphisms (long and triangular face with a prominent forehead), intellectual disability, and behavior problems such as anxiety^32^. Patient 2 presented with severe intellectual disability, a long and triangular face with a prominent forehead, deeply set eyes, exotropia, tooth malposition, dolichostenomelia, pectus excavatum, and arachnodactyly. An axial tomography brain scan showed normal findings (Table 1, Fig. 1F). Additionally, genetic analysis revealed a novel heterozygous *NFIX*: p.[Pro249Metfs36] frameshift mutation, which is consistent with this syndrome. The healthy parents did not consent to be tested (Fig. 2B); however, we presumed a de novo mutation since patients with pathogenic variants in this gene without clinical manifestations have not been reported. To date, no significant differences have been observed in the age of presentation or the number of clinical manifestations associated with *NFX1* point mutations or gross deletions. However, Priolo et al*.* reported a correlation between an 11p13 gross deletion and the occurrence of seizures, which may be explained by a contiguous gene syndrome^32^. Despite these findings, a clear genotype‒phenotype correlation remains difficult in Malan syndrome patients.

Patient 4 presented with a severe ophthalmologic phenotype, including microphthalmos and corneal opacity in the OD and anophthalmia in the OS. Additionally, neurologic, hearing and auricular, dental, and limb manifestations were observed (Fig. 1I–L, Table 1). In this patient, the WES study showed a likely pathogenic heterozygous variant in *FGFR2*, p.(Arg210Gln), that has not been described thus far (Table 2). Mutations in the genes encoding fibroblast growth factor receptor 2 (*FGFR2*), *FGFR3*, and *FGF10* are known to cause autosomal dominant lacrimo-auriculo-dento-digital (LADD1) syndrome. LADD1 clinical signs can vary among individuals, but the condition is characterized by involvement of the lacrimal and salivary ducts or glands (71%), auricular anomalies and hearing deficits (50%), dental manifestations (characterized by caries), and digital anomalies^33^. The patient's 4 phenotypes, characterized by corneal opacity, cup-shaped and low-set ears, severe hearing impairment, conical teeth, and syndactyly, were consistent with *FGFR2*-LADD1 syndrome. Additionally, the facial phenotype observed in the patient's father bore a subjective resemblance to those documented by Inan et al.^34^, and Rohmann et al.^35^. Remarkably, neither short stature nor craniosynostosis was discernible in the patient or among family members, thus negating the possibility of other syndromes linked to *FGFR2* mutations and craniosynostosis. To our knowledge, approximately 50 patients with clinically diagnosed LADD1 syndrome have been reported. Additionally, other features, such as facial dysmorphism^35^, unilateral choanal atresia^34^, joint hypermobility, hypospadias, gross motor delay^36^, and renal abnormalities, may sometimes be present in these patients. Ophthalmologic manifestations in LADD1 syndrome patients are primarily associated with tear duct obstruction, keratoconjunctivitis resulting from inadequate tear production, and corneal erosions. However, Simpson et al. reported a unique case of an LADD1 patient with glaucoma and a PV in the *TP63* gene^37^. Similarly, Inan et al*.* reported another LADD1 patient with ptosis, hypertelorism, and telecanthus, while Rohmann et al*.* described an LADD1 boy with strabismus^34,35^, as well as patients without ophthalmological manifestations. Additionally, Cortes et al. suggested that limbal stem cell deficiency in two patients was related to LADD1^38^. Patient 4 presented with corneal opacity, anophthalmia, and microphthalmia, with the latter two manifestations being described for the first time in this pathology.

**Figure 4:**
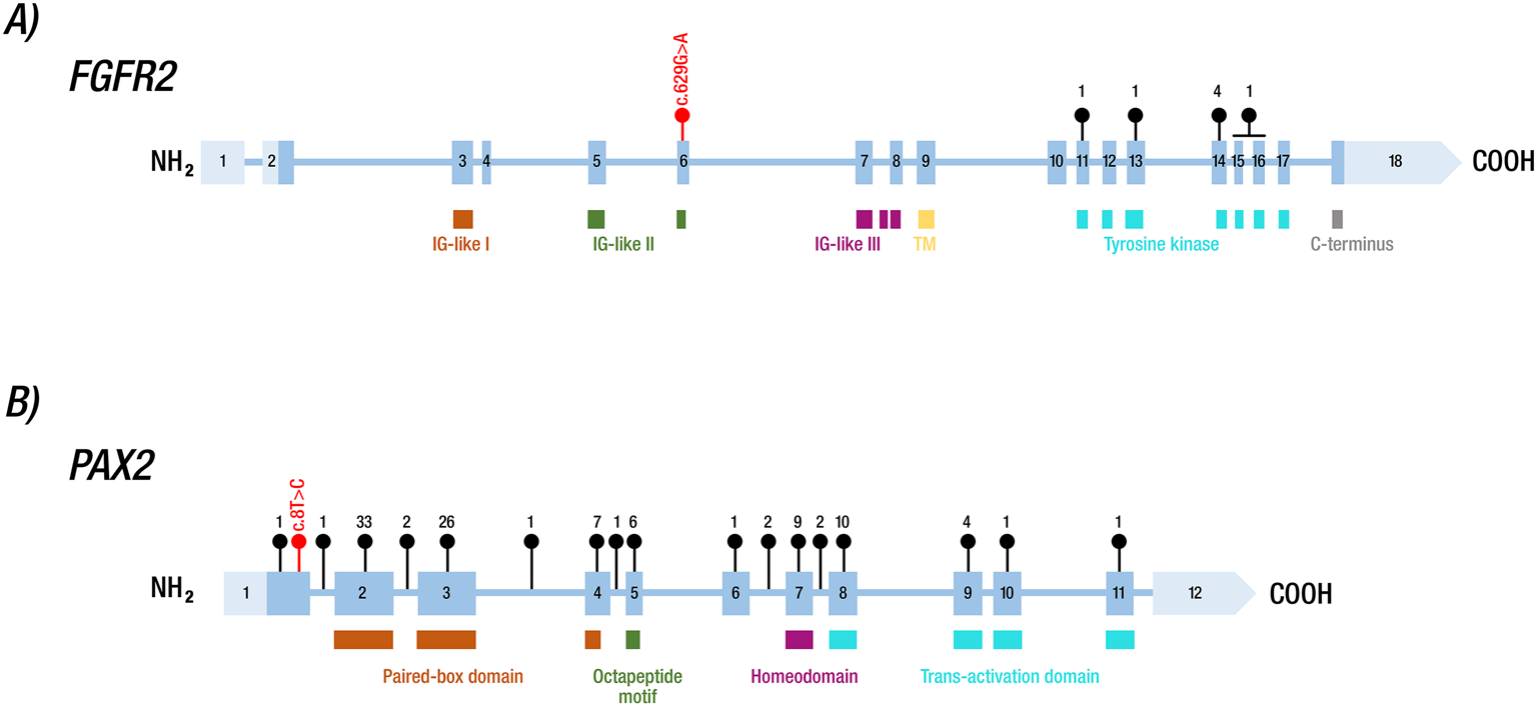
Schematic representation of unique *FGFR2* and *PAX2* pathogenic, likely pathogenic and VUS reported variants. The blue squares represent exons: light blue indicates UTR regions, while dark blue shows the coding sequence. The black numbers inside the blue squares depict the exon numbers. Lines connecting exons indicate intronic regions. Exon‒intron dimensions are not included in the scale measure. The main protein domains are represented below the gene structures. (**A**) Upper numbers show previously reported index LADD1 patients with *FGFR2* mutations in the literature; the c.629G>A variant reported herein is shown in red. (**B**) Upper numbers show previously reported *PAX2* variants reported in the LOVD database v.3.0; the c.8T>C variant reported herein is shown in red. COOH: carboxyl; IG: immunoglobulin; NH2: amino; TM: trans.

Among the 22 published LADD1 patients with a characterized genotype^33,35–37,39–42^, only seven index patients (six families/one novel patient) presented variants in the *FGFR2* gene,^33,35,36,41^ all of which were located in the tyrosine kinase domain (Fig. 4A). In contrast, the FGFR2 strains described herein, c.629G>A or p.(Arg210Gln) LPV, are located at the IG-like II domain, especially in exon 6. This region is highly conserved, with no other variants documented in this area (Figs. 3A, 4A). This could explain the differences in the severity of ocular manifestations observed in Patient 4, highlighting the variable expression of the disease^33–35^ or the influence of modifier genes. Furthermore, the presence of PV in other genes, which were not detected in this WES study, cannot be excluded as a potential explanation for the ocular phenotype associated with microphthalmia and anophthalmia. A whole genome sequencing study could offer additional insights and greater clarity on this matter. Interestingly, the Sanger sequencing revealed that this variant was inherited by his father (Fig. 2D), who presented dysplastic ears, a preauricular tag, and a conical tooth; no ocular abnormalities were found in the father (Fig. 1N). Furthermore, other members of the father´s family, including his paternal grandmother and great aunt, exhibited conical tooth and ear abnormalities (Fig. 2D), but molecular testing could not be conducted on them.

Patient 5 presented a remarkable phenotype characterized by microphthalmia in the OD; microcornea, iris coloboma, and cataract in the OS; optic nerve aplasia; and retinal detachment in the OU (Fig. 1O,P, Table 1). She also presented with neurodevelopmental delay and hypotonia. Cerebral MRI revealed fronto-parietal cortical and subcortical atrophy, as well as a reduction in the optic tracts, chiasm, and optic nerves. The first molecular approach involving SNPs-Array46 (based on CytoScan^®^ technology) performed in this patient revealed no alterations [arr(1-22,X)x2]; therefore, she was a candidate for a WES study. This molecular study revealed a heterozygous allele in *PAX2*, c.8T>C or p.(Met3Thr), which attracted our attention because this gene has been implicated in papillorenal syndrome (PAPRS). PAPRS is a rare autosomal dominant genetic disorder characterized by eye and kidney abnormalities. The most common ocular manifestations in these patients include optic nerve coloboma leading to visual impairment, morning glory anomaly, excavation of the optic disc, an enlarged optic disc with abnormal vasculature, and cilioretinal arteries. Infrequent eye findings include keratopathy, scleral staphyloma, optic nerve cyst, microphthalmia, and pigmentary macular dysplasia. Abnormal renal structure/function is a prevalent finding in *PAX2* patients (92%)^43^. This includes renal hypodysplasia (with fewer nephrons, tissue disorganization and smaller kidneys), multicystic kidney, oligomeganephronia, vesicoureteral reflux, renal hypoplasia and focal segmental glomerulosclerosis^44–46^.

On the other hand, high-frequency sensorineural hearing loss, central nervous system malformations, developmental delay, hyperuricemia, soft skin, ligamentous laxity, and elevated pancreatic amylase are less common manifestations of PAPRS^43,44,46,47^. Notably, *PAX2* phenotypes are tremendously variable, even within the same family^48,49^; thus, establishing a phenotype‒genotype correlation is difficult.

The missense *PAX2* p.(Met3Thr) variant from Patient 5 was classified as a VUS according to the PM2 and PP2 ACMG/AMP criteria. It is located in exon 1, near the DNA-binding domain (paired-box domain), which is a highly conserved region (Figs. 3E, 4B). Although > 50 variants have been described in this domain or in its vicinity (https://databases.lovd.nl/shared/variants/PAX2/unique), only one likely pathogenic mutation (c.5A>G or p.Asp2Gly) was reported in exon 1 (Fig. 4B). This mutation was identified in a patient with congenital abnormalities of the kidney and urinary tract, especially presenting with unilateral ureteropelvic junction obstruction and bilateral vesicoureteral reflux^50^. Since we were uncertain whether the *PAX2* p.(Met3Thr) VUS variant was responsible for the PAPRS phenotype, we modeled the N- terminal region of PAX2 and showed probable structural alterations in the protein, potentially leading to decreased affinity for DNA. PAX2 is a multidomain transcription factor that contains an N-terminal region that binds DNA^50^; therefore, the mutation reported here could promote significant alterations in DNA binding. Since another adjacent probable pathogenic variant has been reported in the same exon 1, and due to the importance of the N-term with respect to DNA binding, we propose that the Met for Thr substitution would potentially have consequences on the affinity for this molecule and consequently on its general functions. Notably, the *PAX2* p.(Met3Thr) variant also occurs at a residue highly conserved among vertebrates (Fig. 3E).

In addition, we performed molecular screening using direct Sanger sequencing in both parents, revealing the maternal origin of the mutation (Fig. 2E). Following the clinical evaluation of the patient's mother, significant kidney findings were noted: she exhibited total protein in a 24-h urine test of 365 mg/24 h (reference values 0–150 mg), but structural kidney ultrasonography did not reveal any abnormalities. Notably, individuals with a normal kidney structure tend to have a PV in the paired domain region (*P* < 0.5)^51^, and the c.5A > G mutation is located near this domain. Unfortunately, a comprehensive ophthalmic examination could not be conducted on the mother due to the lack of patient follow-up. It is important to note that in individuals with pathogenic variants in the *PAX2* gene, the penetrance of eye malformations is at least 77%^43^. This should be regarded as a minimum estimate, as 21% of individuals with *PAX2* pathogenic variants have not undergone a dilated eye examination to evaluate subclinical abnormalities of the optic nerve.

On the other hand, although kidney ultrasonography revealed no abnormalities in Patient 5 at the age of 12 months, the possibility of underlying kidney disease cannot be entirely ruled out, particularly considering the patient's age and the potential for latent manifestations in the future. Additionally, the reported kidney and eye symptoms in PAPRS are diverse, and patients with the same mutation could exhibit different phenotypes^49^.

Likewise, the extracellular portion of the D2 domain of FGFR2 has been shown to interact with its ligand FGF1, which activates signaling cascades, including embryonic growth, cell proliferation, and angiogenesis^52^. Due to its great relevance, the loss or decrease of interactions between FGFR2 and its ligands, including FGF1, could alter such signaling pathways. In the present study, we observed that the Arg210 by Thr mutation, which is located in the extracellular D2 domain of the receptor^53^, leads to the loss of critical interactions that could result in decreased affinity for the FGF1 subunit. Notably, Arg210 has a solvent-accessible surface area of 30.9% (compared to that of the tripeptide Gly-Arg-Gly entirely exposed to solvent)^54^, and it plays a crucial role in interacting with adjacent subunits. Nonconservative mutations in this region have been shown to cause a significant loss of affinity for adjacent subunits, as previously reported^55^. Such alterations in affinity could impact downstream signaling activity, similar to the effects observed when suramin blocks the interaction of both subunits^56^.

Patients with negative WES results, including Patient 6, who presented with corneal dystrophy exhibiting an autosomal dominant pattern, are potential candidates for whole-genome sequencing, as it is important to explore noncoding regions where genetic variants may also be present. In addition, we cannot exclude the possibility of teratogenicity and multifactorial etiology, as suspected in Patient 9, where the mother had a history of gestational diabetes (Supplementary Material).

## Conclusions

WES has been remarkably effective in diagnosing patients who present various systemic alterations with any suspected diagnosis. Moreover, it has demonstrated efficacy in individuals who present phenotypes that may exhibit overlapping features with multiple genetic disorders. This molecular approach has enabled us to achieve a diagnostic detection rate of 40%, thereby characterizing exceptionally rare syndromes. Given that the majority of these syndromes have not been previously documented among Mexican patients, our findings contribute significantly to both the clinical and molecular understanding of these infrequently encountered entities with poorly known prevalence rates. To the best of our knowledge, this study represents the second Mexican report in which WES was used to address ocular diseases. However, our study uniquely included patients who presented with intricate systemic manifestations. This study effectively demonstrated the utility of this molecular technique in patients with ocular disorders accompanied by systemic findings.

The WES approach can be regarded as the gold standard method for identifying the underlying genes responsible for syndromic conditions and exploring their potential implications for future therapeutic interventions. Consequently, this approach significantly diminishes the arduous diagnostic journey encountered by these patients. However, a notable limitation of our study stems from the fact that WES is designed exclusively for analyzing coding regions, which leads to the omission of noncoding regions as well as the potential inability to detect structural or copy number variants. Moreover, variations within regulatory regions were not explored. To overcome these limitations, more extensive investigations, particularly those employing whole-genome sequencing, should be conducted, especially among patients who yield negative results.

Finally, the protein modeling of those variants that were not clearly pathogenic provides us with additional information to propose their potential responsibility for the patient's phenotype, as they might disrupt crucial functional domains within the proteins, possibly compromising their proper functioning. Nevertheless, forthcoming investigations encompassing functional assays are imperative to comprehensively evaluate their biological repercussions and definitively establish their involvement in the disease.

## Methods

### Genomic DNA extraction and PCR

Patients were studied at the Asociación para Evitar la Ceguera en México, Mexico City. This study was approved by the Research Ethics Committee and the Research Committee of the Asociación para Evitar la Ceguera en México and was conducted in accordance with ethical principles and The Code of Ethics of the World Medical Association (Declaration of Helsinki). The parents or legal guardians of each patient signed an informed consent form before performing the molecular study, as well as an iconographic informed consent form for the collection and use of clinical photographs. Total peripheral blood leukocytes were obtained from the 10 patients, their available parents and first-degree affected family members. Genomic DNA was obtained with a commercially available silica-based kit (QIAamp®; Qiagen, Victoria, Australia) according to the manufacturer’s protocol.

### Molecular analyses

Five patients (5/10) underwent cytogenetic studies (karyotyping by GTG-banding and/or SNPs-Array46 750k), and central nervous system imaging was used as the initial diagnostic approach (Table 1). Since none of the ten patients had a clinical or genetic diagnosis, we performed a WES study by the 3billion Laboratory (Seoul, South Corea) using gDNA extracted from peripheral blood samples. All coding regions of the 22,000 human genes were captured by the xGen Exome Research Panel v2 (Integrated DNA Technologies, Coralville, Iowa, USA). The captured regions were further sequenced with a NovaSeq 6000 instrument (Illumina, San Diego, CA, USA) to produce 150 bp paired-end reads. The raw sequencing data were aligned to the GRCh37/hg19 human reference genome using BWA-MEM. Variants were called by GATK v.3. toolkit^51^ and annotated with the Ensembl Variant Effect Predictor^57^. The sequencing data were analyzed using 3billion bioinformatics pipeline evidence as previously described^58^. Estimation of allele frequency, evidence data on the pathogenicity of variants associated with diseases, and transformation of the patient's clinical phenotypes to corresponding standardized human phenotype ontology terms are the algorithms utilized for prioritizing the identified variants, in addition to adhering to the ACMG guidelines. Selected single nucleotide variants and insertion/deletion variants (INDELs) were subjected to automated bidirectional Sanger sequencing (performed by Macrogen, USA)^59^. When we suspected a familial case, we also analyzed available parents and potentially affected family members by bidirectional Sanger sequencing. The primer sequences and amplification conditions used are available upon request.

The pathogenicity of each variant was evaluated according to the American College of Medical Genetics and Genomics and the Association for Molecular Pathology (ACMG/AMP) guidelines^60^. The Mutalyzer nomenclature module tool (http://www.mutalyzer.nl) was used to validate the sequence variant nomenclature of the *GZF1*, *NFIX*, *TRRAP*, *FGFR2* and *PAX2* variants according to the guidelines of the Human Genome Variation Society. The novel variants have been submitted to LOVD v.3.0. (for accession numbers, see Table 2).

### Protein modeling of the two missense FGFR2 and PAX2 variants

Protein modeling was conducted for the two missense variants in FGFR2 and PAX2, aiming to assess their potential structural consequences.

Since the crystallographic structure of FGFR2 in complex with FGF1 is already available in the Protein Data Bank (PDB code: 3OJ2)^61^, it was used to model the corresponding mutation at amino acid position 210: p.(Arg210Gln). It is worth mentioning that such a crystallographic structure corresponds to the N-terminal region of FGFR2 from amino acid residues 151 to 359; therefore, it was used to model the corresponding mutation. Likewise, to obtain a 3D model of PAX2, the crystallographic structure of PAX5 with PDB code 1K78 was used^62^. At the N-terminal region (amino acid residues 16 to 145), PAX2 and PAX5 have 97.7% amino acid identity. Therefore, the crystallographic structure of PAX5 was used to model the N-terminal region of the PAX2 protein, for which the Robetta server was used for molecular modeling^63^. Once we obtained the PAX2 3D model, it was subjected to energy minimization with the Molecular Modeling System UCSF Chimera software^64^. The resulting new atomic coordinates were manually inspected, evaluated for geometric quality, and validated on the MOLPROBITY website (http://molprobity.biochem.duke.edu)^65^. The improved 3D model of PAX2 was aligned with the crystallographic structure of PAX5 (from amino acid residues 19 to 76), both showing remarkable structural similarity (RMSD for all the Cα atoms = 0.39 Å); subsequently, the crystallographic coordinates of the PAX5 protein were removed to analyze the DNA structure (included in the original crystallographic coordinates) in the presence of the PAX2 model. Both the FGFR2 and PAX2 structures were analyzed with the molecular visualizer PyMOL (Molecular Graphics System, v.2.2.0 Schrödinger, LLC).

### Supplementary Information

Supplementary Figure 1.

## Data Availability

All identified variants were submitted and are available at the Leiden Open Variation Database; LOVD v.3.0. (http://www.lovd.nl/GZF1, http://www.lovd.nl/NFIX, http://www.lovd.nl/TRRAP, http://www.lovd.nl/FGFR2 and http://www.lovd.nl/PAX2), under the following accession numbers: GZF1_00402795, NFIX_00402809, TRRAP_00402812, FGFR2_00402813, and PAX2_00402842.
