# Supplementary material for: Deciphering the etiology of undiagnosed ocular anomalies along with systemic alterations in pediatric patients through whole exome sequencing
Source: Sci Rep. 2024 Jun 22;14:14380. doi: 10.1038/s41598-024-65227-6 (PMC11193775; doi:10.1038/s41598-024-65227-6)

Supplementary Material. Pedigrees of five patients with negative WES results



1. Patient 6



1. Patient 7



1. Patient 8
2. Patient 9


3. Patient 10


Supplement: Supplementary file 1 — Supplementary Figure 1. [file 41598_2024_65227_MOESM1_ESM.docx]
